# Effect of power training on function and body composition in older women with probable sarcopenia. A study protocol for a randomized controlled trial

**DOI:** 10.1371/journal.pone.0313072

**Published:** 2025-01-06

**Authors:** Luis Polo-Ferrero, Susana Sáez-Gutiérrez, Arturo Dávila-Marcos, Fausto J. Barbero-Iglesias, María C. Sánchez-Sánchez, Ana Silvia Puente-González, Roberto Méndez-Sánchez

**Affiliations:** 1 Department of Nursing and Physiotherapy, University of Salamanca, Salamanca, Spain; 2 Institute of Biomedical Research of Salamanca (IBSAL), Salamanca, Spain; University Hospital of Padova, ITALY

## Abstract

**Background:**

Sarcopenia is a clinical syndrome characterized by the loss of muscle mass and strength. Hormonal changes that occur early in women may influence protein synthesis and promote muscle atrophy, leading to probable sarcopenia, defined as a loss of muscle strength without an obvious decrease in muscle mass. Various types of exercise have already proven effective in treating sarcopenia. Power training (PT), a subtype of resistance training performed at high speed, has not yet been studied in this population group.

**Methods:**

A randomized controlled trial will be conducted with three parallel groups: a PT group, a multicomponent training (MT) group, and a no-exercise group. The inclusion criteria will be women over 65 years of age who meet the diagnostic criteria for probable sarcopenia (Hand grip test less than 16 kg and Five Times Sit to Stand Test more than 15 s) according to the European Working Group on Sarcopenia in Older People (EWGSOP2). Two assessments will be conducted at the beginning and at the end of the 32 weeks of intervention, in which variables of body composition (muscle, fat and weight) and functionality (strength and physical performance) will be collected. There will be 3 sessions of 50 minutes per week in each intervention group. The masking strategy will be double-blind. The analysis of intergroup differences will be conducted using multivariate and univariate analysis of variance (MANOVA and ANOVA), as well as pairwise comparisons (Bonferroni corrections). Changes in the degree of sarcopenia and how many women eliminate the risk of sarcopenia (no probable sarcopenia) after the different interventions will be tested.

**Discussion:**

The study aims to investigate the impact of PT in older women at risk of sarcopenia. The expected results are that PT will significantly improve functionality and body composition compared to other forms of exercise (MT) and no physical activity. The results may represent a significant advance in understanding and addressing sarcopenia before it becomes established, providing preventive treatment and new tools whose clinical applicability has been studied.

**Trial registration:**

The clinical trial was prospectively registered at ClinicalTrials.gov Identifier: NCT05870046.

## Background

The process of ageing is a biological phenomenon that results in increased susceptibility to disease and a decline in functional capacity [1]. This phenomenon has resulted in a notable increase in the proportion of the population that is ageing, driven by rising life expectancy and declining birth rates [2]. The concept of healthy ageing is defined by five dimensions: metabolic and physiological health, physical function, cognitive function, psychological well-being and social well-being. Among these dimensions, physical function is closely associated with sarcopenia [3].

Sarcopenia is a clinical syndrome characterised by the loss of muscle mass and strength [4]. Aging causes an estimated 1–2% annual loss of muscle mass starting at age 50 years, with a total loss of 45% by age 80 years [5]. The onset of hormonal changes that result in muscle atrophy occurs earlier and progresses more gradually in women than in men. The loss of muscle mass and function, leading to postmenopausal sarcopenia, may be attributed to the gradual decline in oestrogen and androgen levels [6]. This reduction may result in alterations to the secretion of growth hormone and insulin-like growth factor 1, as well as an increase in pro-inflammatory cytokines that promote sarcopenia [7–9]. Furthermore, the decline in progesterone, which plays a pivotal role in regulating the menstrual cycle, also contributes to the development of sarcopenia in older women [10]. These hormonal changes render women more susceptible to sarcopenia at an earlier age than men, whose hormonal decline typically occurs around the age of 80 [11].

Sarcopenia is associated with an increased risk of falls, fractures, physical disability, and mortality [12]. The International Classification of Diseases (ICD-10: M62) formally recognises it as a muscle disease [13]. Sarcopenia is a geriatric syndrome frequently found in older adults, with a worldwide prevalence ranging from 10% to 27% [14]. In recent years, various international groups have published diagnostic criteria for sarcopenia, all using the same tools and tests, but with different cut-off points [15–19]. In Europe, the guidelines recommended by the European Working Group on Sarcopenia in Older People 2 (EWGSOP2) are generally followed. Probable sarcopenia is defined as loss of muscle strength without obvious loss of muscle mass (>5.5 kg/m2 in women and >7 kg/m2). In women, probable sarcopenia is established if the hand grip test (HG) value is less than 16 kg and it takes more than 15 seconds to perform the five times sit-to-stand (5STS) test [15].

Scientific evidence has shown that exercise is currently the main intervention available to combat sarcopenia [20]. A systematic review has shown that different modes of physical training can improve sarcopenia in older adults [21]. The most researched types of exercise are MT and resistance training (RT). Although it has been shown that RT may be more effective than MT in the prevention of muscle loss and functionality, MT also shows good benefits being one of the most practiced types of exercise by older adults [21–23]. RT stimulates satellite cell proliferation and protein synthesis, which are important for maintaining muscle homeostasis in adults with sarcopenia [24]. However, there are different subtypes of RT, such as power training (PT), which involves high contraction speeds.

In healthy older adults PT has been shown to have a greater impact on improving muscle power and physical performance compared to RT. Additionally, it has been shown to reduce the incidence of falls, demonstrating superior effects on functionality than RT in older adults, including strength variables that diagnose probable sarcopenia [25, 26]. However, the effect of PT on older women in the preclinical stage of the disease (probable sarcopenia according to EWGSOP2) has not yet been investigated.

### Aim

In line with the above, the main objective is to assess and analyse the effect of a muscle PT program on muscle condition and degree of sarcopenia, evaluating muscle strength, body composition and functionality in women over 65 years of age with sarcopenia probable, comparing it with a MT intervention and women who do not perform physical exercise.

## Material and methods

### Design and setting of the study

**Fig 1:**
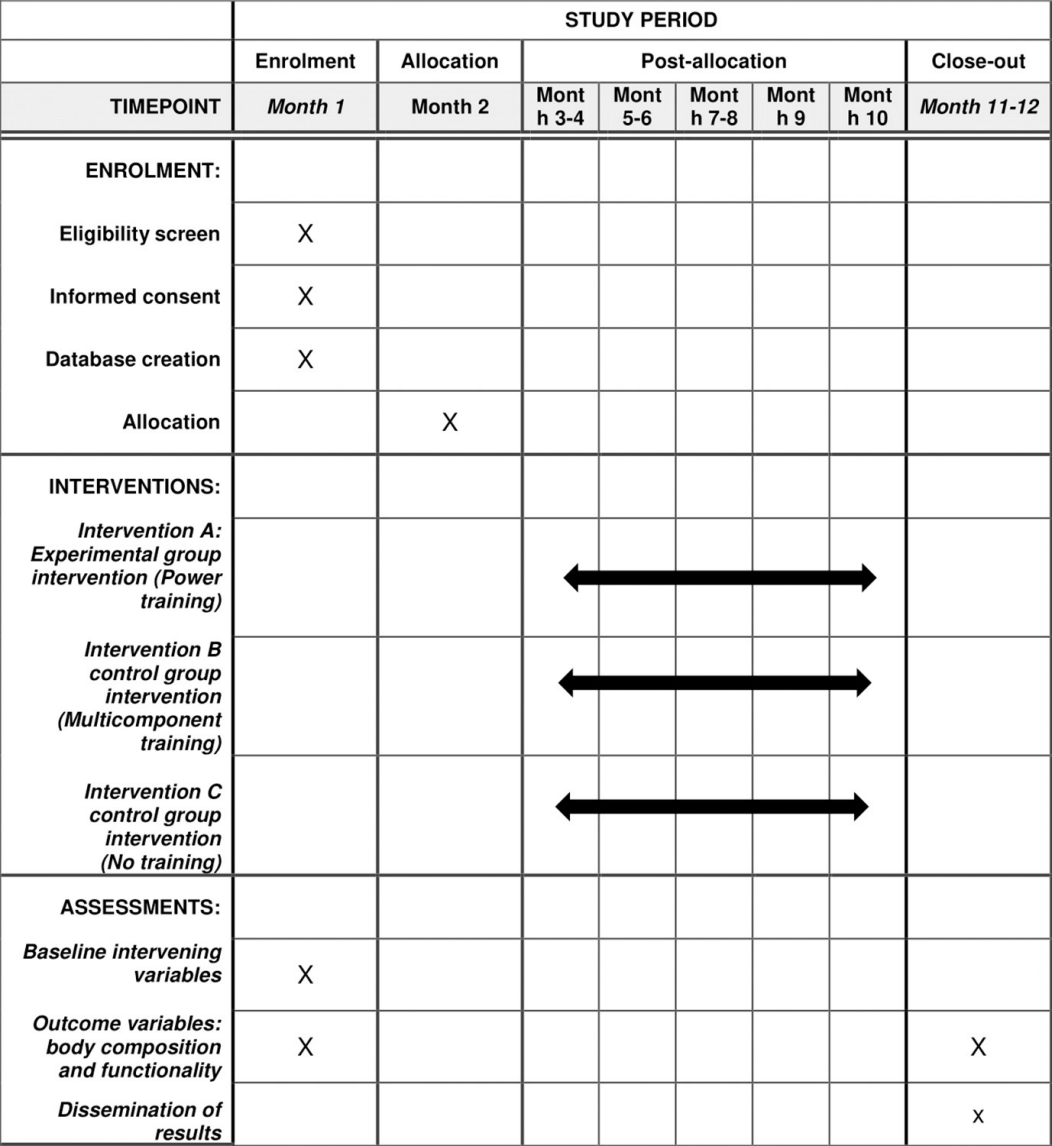
The schedule of enrolment, interventions, and assessments of SPIRIT 2013.

A 1-year, prospective, longitudinal, parallel-group, randomized controlled trial will be conducted. Two experimental groups PT group, MT group and a non-exercise control group will be formed. The reporting of this clinical trial conforms to the Consolidated Standards for Reporting Trials (CONSORT 2010) statement [27]. The development of this clinical trial protocol follows the standards of the SPIRIT statement (Standard Protocol Items: Recommendations for Interventional Trials) (S1 Table) [28]. The schedule of enrolment, interventions and assessments as detailed in Fig 1 following recommendations of the SPIRIT 2013 Statement (Fig 1)

The protocol for this trial received approval from the Salamanca Health Area Drug Research Ethics Committee with code PI 2023061317 and was conducted in accordance with the Declaration of Helsinki [29]. The clinical trial was registered in ClinicalTrials.gov (NCT05870046).

All participants will be members of the Geriatric Revitalization Program (GRP) and will exercise at their respective senior centres. Selection criteria will be applied to determine eligibility. Eligible participants from the same senior centre will be grouped together and randomly assigned. As the sessions are held in different centres in Salamanca, only 5 to 15 participants will be allowed to take part in each centre.

The randomization will be performed using R software version 4.2.1 by an external statistician who will not participate in the analysis.

**Fig 2:**
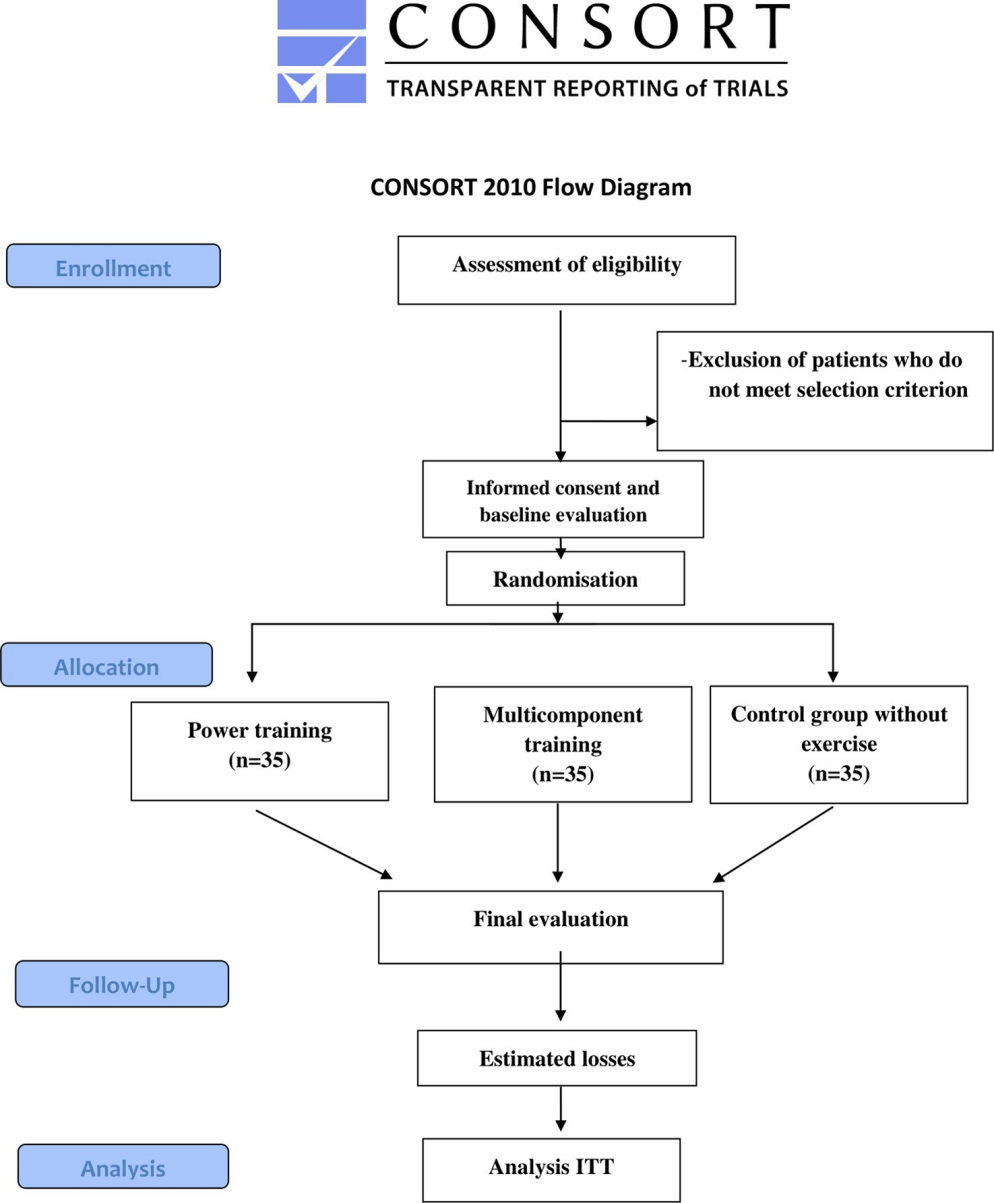
Study methods flow chart.

Blinding will be based on the study hypothesis and the participants assigned to each group. The strategy employed will be double-blinding. Both the assessors and the statistical researcher will be blinded. The external researcher responsible for randomization will inform each instructor which intervention to administer in each group. The evaluators will not know the assignment of the groups and that the confidentiality of the identification of the participants and their codes will be maintained. The researchers who will teach the exercise sessions will not participate in the evaluations of the study outcome variables. The participants will know the type of intervention due to its nature, but they will not know what type of exercise or the study hypothesis. Blinding will not be disclosed under any circumstances. The study methods flow chart can be seen in Fig 2.

### Sample/participants

#### Participants

All participants who voluntarily sign up for the GRP will be required to read and sign the informed consent form (See S1 File). The GRP is a project of the Faculty of Nursing and Physiotherapy at the University of Salamanca that offers older people in the city of Salamanca the opportunity to engage in physical exercise. Only those who meet all the selection criteria will be included.

The criteria for participation in the GRP are as follows: being female, over 65 years of age, residing in the community of the city of Salamanca, and meeting the diagnostic criteria for probable sarcopenia according to the EWGSOP2.

Exclusion criteria for participation in the study include previous participation in another exercise program, presence of fibrillations, uncontrolled cardiac pathology or hypertension, pacemaker use, history of severe trauma or recent surgery, congenital collagen involvement, severe mental illness, and any other circumstance that may interfere with the purpose or development of the study, as determined by the investigators. Elimination criteria during the intervention will include total attendance less than 85%, participation in other exercise programs during the intervention period, and changes in physical activity level outside the program.

#### Sample size

Sample size was calculated for the main study variables, 5STS and HG. The minimum detectable change in similar populations and interventions was 2.5 points (a decrease in 5STS and an increase in HG) to consider a real change beyond measurement error [30, 31]. The study used GRANMO version 7.12 of April 2012. Assuming three groups, with an alpha risk of 0.05 and a beta risk of 0.2 in a bilateral contrast. A loss-to-follow-up rate of 20% was estimated, given that the intervention was extensive and older people are known to have lower adherence to physical activity programs [32]. It was concluded that 105 subjects would be needed among the three groups with a 1:1:1 ratio to detect a difference equal to or greater than 2.5 units, assuming a standard deviation of 5 units.

### Procedures and data collection

#### Evaluations

Two evaluations will be conducted at the Faculty of Nursing and Physiotherapy of the University of Salamanca. The baseline assessment will take place after recruitment and before randomisation and allocation of subjects to the corresponding group. The preliminary assessment documents the independent variables and the primary outcome variables of the study. Intervention variables will also be recorded in detail and various objective tests will be applied. Subsequently, after randomization, different interventions tailored to each group will be carried out.

The final evaluation will be conducted at the end of the intervention period with no time intervals between evaluations. All tests will be performed by the same investigator in a standardized manner, as detailed in S2 Table. Investigators will be trained and have more than three years of experience in geriatric assessment. Results will be communicated through individualized, personalized reports to individuals who have indicated a preference to receive updates on results and progression at the outset.

#### Description of the variables

After signing the informed consent, a clinical history and a functional and body composition assessment will be performed for everyone, regardless of the group to which they are assigned. Sociodemographic variables, variables examining functionality (which include the two main study variables) and body composition variables will therefore be recorded. All of these are detailed below:

1. **Sociodemographic variables**: personal data, medical history, presence of comorbidities and concomitant medication use will be collected. At the end of the interview, blood pressure will be taken.
2. **Primary variables**: the tests used to establish that the person has probable sarcopenia or sarcopenia probable according to the EWGSOP2 will be chosen:
  - Five-Repetition-Sit-To-Stand Test (5STS): this is a functional test that assesses a person’s ability to stand up and sit down from a chair for five times without using the arms (lower extremity strength). The 5STS has been shown to be a more sensitive and specific measure than the original test [33]. It also offers excellent test-retest reliability (0.89) [34].
  - Hand grip test (HG): is a quick, easy and inexpensive test that measures hand grip strength. This test is recognized in clinical practice and in the literature as a standard instrument for measuring HG [15]. It will be performed with the Jamar Plus device in standing position and with the elbow flexed to 90 degrees, which has been shown to have excellent test-retest reproducibility (r>0.80) and excellent inter-observer reliability (r = 0.98) [35, 36].
3. **Secondary functional variables**: the Sarc-f questionnaire will be administered as a rapid diagnostic test for sarcopenia that predicts the need for further evaluation [37]. Physical activity will be monitored through the International Physical Activity Questionnaires (IPAQ) [38]. The Short Physical Performance Battery (SPPB), as it is a rapid, objective three-test physical function test that measures lower extremity strength, gait speed, and balance [39]. The Timed Up&Go (TUG), which is a predictor of frailty and falls, widely used in geriatric research to measure balance and mobility in older people [40]. Gait speed, which is a simple, objective and valid measure of human gait performance that has been used in a variety of settings and is recommended for use as a "vital sign" of health [41]. Two-minute step test to measure aerobic capacity in older adults [42]. The number of falls will also be recorded one year before the start and during the intervention.
4. **Secondary body composition variables**: electrical bioimpedance (TANITA BC-418) will be used to measure most body composition parameters. The total percentage of fat (BF%) and muscle, which is important for general health, will be calculated [43]. Additionally, appendicular muscle mass (ASM) will be calculated, as it is one of the most important variables in the diagnosis of sarcopenia today. It is generally adjusted for height to obtain the appendicular muscle mass index (SMI) (ASM/height^2^). In order to predict the risk of developing chronic diseases, measurements of visceral fat, waist circumference (WC), and body mass index (BMI) (weight/height^2^) will be taken [15]. Height will be measured with a height rod.

All these outcome variables will be measured at baseline and at the end to check the change after the different interventions.

#### Interventions

All study participants will continue with their medical treatment guidelines, level of physical activity, and dietary pattern. Two parallel intervention programs will be designed; the control group will not receive any intervention. The exercise sessions, which will be conducted by healthcare professionals with more than one year’s experience in providing exercise sessions for older adults, will be held at senior centres throughout the city. Adherence to the program will be verified by daily attendance at exercise sessions.

The PT and MT sessions will follow the ACSM recommendations and the basic principles of the FITT-VP (frequency, intensity, type of exercise, time, volume and progression) are followed [44]. There will be three 50-minute sessions per week for 32 weeks. The sessions will begin and end with common exercises, starting with active mobility and ending with stretching and diaphragmatic breathing. The load progression will be based on individual perceived exertion. At the end of each week, participants will be asked to rate their perceived exertion using the modified Borg scale. If the score is less than 7, the intensity of the exercises will be increased for the following week to maintain a consistent level of 7 on the scale. The experienced health personnel delivering the sessions will determine how to progress, such as increasing external weight, repetitions, sets, reducing rest time, or performing unilateral exercises. Although these are group sessions, progression and volume will be adjusted on an individual basis, due to the variability of comorbidities that older adults may present.

The rooms in the senior centres for the interventions will maintain a uniformity in their infrastructure, ensuring in all cases an environment conducive to the optimal realization of the intervention sessions. To encourage adherence, attendance will be collected for each session. If they do not attend, they will be called to their attention, even warning that they may be excluded. Although the number and duration of sessions will be identical in both groups, the nature of the intervention will be different for each study group, and participants will remain unaware of their assignment to a specific group.

- **Power training (PT):** The objective of this group is to perform the strength exercises at high speeds. To avoid and minimize the possible risks that older adults may have and to have 8 months of intervention, a correct progression of the load will be carried out. A series of simple exercises with little material will be proposed. They will be based on lower limb exercises (squat, deadlift, front and sagittal lunges, hip extensions and abductions and heel raises) and upper limb exercises (wall push-ups, arm raises, elbow push-ups and extensions). We have tried to design a simple and low-cost exercise program with the objective that the participants will continue to perform it once they finish the study. The exercises, the phases of the program, the objectives and the ways to progress can be seen in Table 1. The exercises, the phases of the programme, the objectives and the ways of progressing can be seen in Table 1.
- **Multicomponent training (MT)**: it will include different modalities of moderate-intensity physical activity. During the session, aerobic, active mobility, strength, balance and coordination exercises will be integrated as shown in Table 2.
- **Control group (CG):** participants in this group should continue with their usual dietary pattern and level of physical activity, without modifying their lifestyle habits during the study period. They will not participate in any of the two programmes, they will not be able to do physical exercise in a systematic and programmed way in any other programme.

**Table 1:** Exercise and phases of the power exercise program.

**UPPER LIMB EXERCISES**			**LOWER LIMB EXERCISES**	
Wall push-ups, arm raises, elbow push-ups and extensions.			Squat, deadlift, front and sagittal lunges, leg extensions, hip extensions and abductions and heel raises.	
**Target phase**	**Objetives**	**Types of contraction**		**How to progress**
**1st phase: Adaptation to the exercise (1st month)**	Teach correct execution of resistance exercise patterns.	Isometric exercises and very controlled isotonic contractions.		Increase isometric contraction times, range of motion, remove supports, etc.
**2nd phase: resistance training (2nd-3rd month)**	Progress in resistance exercises.	Isotonic exercises (concentric, eccentric) at low and normal speeds.		Increase the number of repetitions per set, the number of sets or the external weight, progress towards unilateralism, decrease rest time between sets, etc.
**3rd phase: high-speed resistance training (4th-8th month)**	Gain muscle power.	Isotonic exercises progressively increasing the speed of the concentric phase.		Increase the speed of the concentric contraction and the number of repetitions at maximum speed. Subsequently, the number of series will be increased, external weight will be added or progress towards unilateral at maximum speed.

**Table 2:** Sample session of the principal part of multicomponent training.

EXERCISE COMPONENT	EXERCISES	FORM OF PROGRESSION
**AEROBIC EXERCISE 10´**	Walking, running.	Increases in continuous walking speed, progress to running start, increases in pace, intervallic work, progressive decrease in rest.
**RESISTANCE EXERCISE 10´**	Upper and lower limb isotonic exercises.	Increase number of repetitions per set, number of sets, progress towards unilateral, decrease rest time between sets.
**COORDINATION AND BALANCE 10´**	Balance exercises, dual task and coordination of upper and lower limb.	Progress by disturbing vestibular system, visual, monopodal support, imbalances, unstable surface, complication of dual-task exercises, more complicated and faster decision making, etc.

### Data analysis

Statistical analysis will be performed using an intention-to-treat (ITT) approach. Baseline differences between groups will be tested using normality plots and the Kolmogorov-Smirnov test. Homogeneity between groups will be assessed using Levene’s test, thus ensuring successful randomisation. Missing data will be handled using the imputation method of the last observation carried forward. It will be decided to keep outliers in the analysis, as they may represent legitimate outliers or relevant information, which will be discussed in the future study.

A descriptive analysis of the dependent and independent variables of each group will be carried out. Quantitative variables will be expressed as mean ± standard deviation, while qualitative variables will be presented as frequency distributions and percentages.

To assess the effect of the interventions on the study variables in each group, multivariate analysis of variance (MANOVA) will be used as the main method. The variables will be grouped into two large blocks: those measuring functionality (HG, 5STS, GS, TUG, Two-minute step test and SPPB) and those measuring body composition (weight, WC, BF%, visceral fat, ASM, SMI and BMI). Subsequently, MANOVA will be performed on the difference in means after the intervention, using time as an intra-group factor and type of intervention as an inter-group factor. To graphically represent the MANOVA, the canonical Biplot will be displayed, including the confidence circles representing the set of variables in each intervention group. If the circles do not overlap, it can be concluded that there are significant differences between the groups.

To verify the results of the MANOVA, differences between groups for each quantitative variable, both functional and body composition, will be analysed using the repeated measures univariate analysis of variance (ANOVA) test for normal and homogeneous variables or the Kruskal-Wallis test for non-normal or heterogeneous variables. As an additional method, pairwise comparisons will be performed using Bonferroni’s test for normal and homogeneous variables and Dunn’s test for non-normal and heterogeneous variables to determine between which groups there are significant differences.

The effect size in ANOVA of the results obtained in each group will be measured by eta squared (η^2^). Values around 0.01 indicate a small effect size, around 0.06 a medium effect size, and 0.14 or more a large effect size.

A 95% confidence interval will be used to establish any differences, and statistical significance will be reported for all differences with p < 0.05 (bilateral). All statistical analyses will be performed using IBM SPSS for Windows (version 26.0, Armonk, NY. IBM. Corp.).

### Ethical aspects

The study was approved by the Ethical Committee for Research with Medicines of the Salamanca Health Area with the code PI 2023061317, having obtained the prior written informed consent of the study subjects and in conformance with the Helsinki Declaration [29].

The participants, before the start of the study, will be informed of the objectives of the project and the risks and benefits of the explorations that will be carried out; subsequently, they must sign the informed consent. In the carried out way, the confidentiality of the subjects included in the study will be guaranteed at all times, in accordance with the provisions of Organic Law 3/2018, of December 5, on the Protection of Personal Data and guarantee of digital rights, under the conditions established by Law 14/2007, of July 3, on biomedical research and Regulation (EU) 2016/679 of the European Parliament and of the Council, of April 27, 2016, on Data Protection (RGPD). Data and information will not be shared with third parties. Each participant will be coded, so their personal data will not be known. Only those who perform the statistical analysis will have access to the database.

Significant modifications to the protocol (such as changes in the tools of evaluation, modifications to the selection criteria or to the interventions) will be communicated immediately to the Ethics Committee.

And since this is a randomised clinical trial, it follows the CONSORT guidelines, and it was registered. TRIAL REGISTRATION: ClinicalTrials.gov; ID: NCT05870046.

### Rigour

This study has been developed in accordance with the recommendations supported by the SPIRIT Declaration 2013 regarding the essential content of a clinical trial protocol (see S1 Table). Furthermore, the study design follows the guidelines established by the CONSORT 2010 guidelines for the execution of parallel group randomized controlled clinical trials.

For effective description and replication of the proposed intervention, the TIDieR checklist is included as an additional tool (see S3 Table).

Upon completion of the study, the data collected here will be made available to those investigators who request it through FAIR sharing of research data repositories at https://www.re3data.org/.

### Human and material resources

The personal resources are a total of 18 people belonging to the project linked to the GRP. The necessary and adequate spaces for carrying out the evaluation visits and intervention sessions will be the Faculty of Nursing and Physiotherapy of the University of Salamanca and the Centers for the Elderly of the City of Salamanca dependent on the Salamanca City Council.

The material resources used during the study are stopwatches, tape measures, furniture, blood pressure monitor (OMRON® M7 Intelli IT, HEM-7361T-EBK), measuring rod, hand dynamometer (Jamar® Plus Hand Dynamometer), body composition bioanalyzer (Bioimpedance meter TANITA® BC-418), linear encoder (ADR® Encoder), SARC-F questionnaires, material for the exercise sessions (cones, ladders and weights) and IBM SPSS software statistics v. 28 .0

## Discussion

The study aims to investigate the impact of PT on older women at risk of sarcopenia. It is hypothesized that PT can reverse the probable sarcopenic state in women over 65 years of age, acting as an effective preventive therapy that eliminates the risk of sarcopenia and prevents its associated factors. Additionally, it is hypothesized that PT will significantly enhance functionality and body composition in comparison to other forms of exercise and no physical activity.

It is important to note that sarcopenia, which is commonly associated with frailty, is also independently linked to an increased risk of falls [45, 46]. By preventing sarcopenia, hospitalizations, institutionalizations, and other associated adverse effects can be indirectly avoided. The scientific community has demonstrated with high certainty that exercise programs reduce the rate and number of falls in the elderly [47]. Therefore, we expect both PT and other forms of exercise to improve this aspect, with a particularly optimistic expectation for PT.

Meta-analyses have been conducted to evaluate the effect of PT on healthy older adults [25, 26]. Similarly, meta-analyses have been performed to assess the impact of RT on function and body composition in people with sarcopenia [15, 48, 49]. However, this study is the first of its kind to investigate older women with probable sarcopenia according to EWGSOP2 diagnostic criteria. This study aims to address a gap in scientific knowledge by investigating an understudied area. To our knowledge, only one other clinical trial has evaluated the effect of RT in older women and men with probable sarcopenia. However, this trial only assessed appendicular lean mass and BF% [50].

This study investigates the effect of PT as a preventive treatment in older women with probable sarcopenia. It represents an important advancement in understanding and addressing this condition, as scientific evidence on the various forms of preventive treatment available for sarcopenia has been insufficient. However, this study’s findings will not only fill the knowledge gap but also support the recommendations issued by the EWGSOP2 in 2019. This will provide a solid basis for implementing effective prevention and treatment strategies for sarcopenia in older women [15].

In addition, it is important to consider that the PT exercise program in this study is designed in such a way that any older adult can carry it out at home safely, without requiring any material, and the progression of the load is based on the perception of effort. Therefore, at the end of the program, people will be able to continue doing it at home without the need to calculate the MRI or incur additional expenses. This aspect is of great importance, as it facilitates the transfer of research to society, allowing older adults to benefit from the potential effects of power training when they are in states of sarcopenia probable.

### Limitations

The study protocol has limitations that should be communicated to inform the interpretation of the results and the design of future research. Although the study follows all CONSORT recommendations, participating subjects will not be blinded to the intervention due to the nature of the intervention. Given the variability of the factors that may affect this population group, not all of them will be assessed, including dietary patterns and different diseases such as diabetes. The expected results cannot be extrapolated to the general population, to other population groups (such as hospitalized or institutionalized older adults) or to different environments or city. This sample was chosen because older women, the population group that lives in the community, probably represent the most people who may be at risk of sarcopenia. We have prioritized obtaining results with a homogeneous sample over generalizing them to the general population.

Progression during the intervention will be based on each participant’s perception of effort, which may introduce biases as some participants may overestimate or underestimate their effort. Although the physical therapist will instruct participants on how to determine their perceived exertion, it is important to note that their unfamiliarity with this method may affect the results.

### Dissemination plan

The results of this clinical trial will be published in high impact journals of different specialties with the intention of achieving maximum visibility. Knowledge will also be transferred to society through conferences for older adults and dissemination through social networks and the media.

### Future perspectives

The project has the potential to bring practical benefits and transfer results to clinical practice in physiotherapy. It could also have a positive impact on general health, as well as the economic and social spheres. The effect of PT should be investigated in more population groups (men, confirmed or severe sarcopenia) to be able to generalize the results to the general population.

In recent years, there has been increasing interest in studying sarcopenia and aging, including diagnosis and possible interventions to improve this condition. It is widely accepted that a healthy lifestyle, including a balanced diet and regular exercise, is essential to mitigate the negative effects of clinical conditions. The aim of this study is to investigate the effectiveness of PT and MT in improving functional and body composition parameters as a means of preventing sarcopenia.

In 2019, EWGSOP recommended that healthcare professionals take steps to promote early detection and treatment of sarcopenia in patients at risk. It is recommended that the diagnosis, treatment, and prevention of sarcopenia be included in routine clinical practice. Sarcopenia is becoming a major problem for the National Health System, leading to increased healthcare costs due to the aging population. Therefore, it is important to promote cost-effective interventions, such as PT or MT that can reduce these costs and improve the quality of life for those at risk.

## Conclusions

The results of this study protocol may expand treatment options for women over 65 with probable sarcopenia, leading to improved functionality and body composition. Most research has focused on people with confirmed sarcopenia, but only one study has analyzed the effect of RT in this population. This clinical trial is significant because it is the first to evaluate the effect of PT as a preventive measure for sarcopenia in older women, a recommendation endorsed by the European Working Group on Sarcopenia in Older People (EWGSOP2). Prevention of sarcopenia at an early stage is essential to mitigate its adverse effects and improve long-term quality of life in this vulnerable population. The study has the potential to provide strong evidence of the efficacy and feasibility of PT as a preventive intervention. This could have important implications for clinical practice and public health policy. In addition, this study focuses on older women at a stage when sarcopenia is likely to occur. It identifies opportunities for early intervention that could slow or even prevent progression to sarcopenia. This could have a significant impact on the health and well-being of this at-risk population.

In summary, this clinical trial could broaden the therapeutic options available to treat sarcopenia in older women, potentially leading to more effective and personalized interventions to prevent the development of sarcopenia. These findings could contribute to scientific knowledge in this area and have direct implications for improving the health and quality of life of older women worldwide.

## Supporting information

S1 File Informed consent and participant information sheet. (PDF)

S1 Table SPIRIT 2013 checklist: Recommended items to address in a clinical trial protocol. (DOCX)

S2 Table Standardized measurement of the result variables. (DOCX)

S3 Table TIDieR checklist. (DOCX)

## Abbreviations

• 5STS: Five-Repetition-Sit-To-Stand Test
• ACSM: American College of Sports Medicine
• ANOVA: Analysis of variance
• ASM: Appendicular Skeletal Muscle Mass
• CONSORT: Consolidated Standards Of Reporting Trials
• EWGSOP: European Working Group on Sarcopenia in Older People
• CG: Control group without exercise
• GRP: Geriatric Revitalization Program
• HG: Hand Grip Test
• ITT: Intention-to-treat analysis
• MANOVA: Multivariate analysis of variance
• MT: Multicomponent training
• PT: : Power training
