# Supplementary material for: Effect of power training on function and body composition in older women with probable sarcopenia. A study protocol for a randomized controlled trial
Source: PLoS One. 2025 Jan 6;20(1):e0313072. doi: 10.1371/journal.pone.0313072 (PMC11703083; doi:10.1371/journal.pone.0313072)
Supplement: S1 File — (PDF) [file pone.0313072.s001.pdf]

INFORMATION on the EFFECT OF POWER TRAINING ON FUNCTION AND BODY COMPOSITION IN OLDER WOMEN WITH PROBABLE SARCOPENIA.

Principal Investigator: Dr. D. Roberto Méndez Sánchez. University of Salamanca

INTRODUCTION

With this document we would like to inform you about a research study in which you are invited to participate. Your participation is voluntary. Please take as much time as you need to read the following information and refer to it as you wish. Ask the investigator of this study if there is anything that is not clear to you or if you would like more information.

STUDY OBJECTIVES

1. To compare the effect of a strength-based therapeutic exercise program based on muscle power with a mixed training program and to check whether it improves muscle condition and degree of sarcopenia, according to parameters of functionality, strength and body composition in women over 65 years of age with probable sarcopenia.
2. To characterize and describe the muscular state and degree of sarcopenia in a population of women over 65 years of age according to strength, electronic bioimpedance and functional parameters.
3. To analyze the baseline and post-intervention associations and correlations between muscle status and/or degree of sarcopenia (strength, body composition, functionality) with the level of physical activity, nutritional status, degree of frailty and quality of life in women over 65 years of age.
4. To assess the effect of an intervention with therapeutic physical exercise of strength, based on muscle power, versus an intervention with multimodal therapeutic physical exercise (mixed) and the lack of physical exercise (control group), according to the study groups.

METHODOLOGY

This is a Randomized Controlled Clinical Trial with three parallel groups, in which women older than 65 years with pre-sarcopenia grade will be recruited according to the algorithm proposed by the 2019 EWGSOP2. Study participants will be assigned to one of the three study arms: 1) Mixed Exercise Group (MEG); 2) Muscle Power Exercise Group (MEG); 3) No Exercise Control Group (CG). The study will have a duration of 1 year with a 32-week intervention with three weekly 50-minute sessions. There will be 3 evaluation visits, one pre-intervention (baseline visit) and two post-intervention visits at 8 months and one follow-up visit at 12 months. Both the participants and the evaluating investigators will be blinded.

At the beginning and at the end of the study, participants will freely and voluntarily provide the following information and perform the following types of tests and assessments:

1. affiliation or personal data and medical history.
2. Physical condition assessment tests:
   a. Determination of functionality: The following tests will be performed: Timed Up&Go (TUG), SPPB (Short Physical Performance Battery), stair test and walking speed test.
   b. Determination of muscle strength: The following tests will be performed: manual grip strength, 5 squat test, and the speed of execution of the concentric phase of the squat will be assessed (estimation of muscle power).
   c. Determination of body composition: The following will be obtained: weight, Body Mass Index, abdominal perimeter, percentage of body fat and muscle mass, degree of visceral fat, appendicular muscle mass and appendicular muscle mass index adjusted for height.

PERFORMANCE OF THE TESTS AND ASSESSMENTS

All these assessment tests will be administered by a trained and certified physiotherapist evaluator, in optimal hygienic and safety conditions, using approved equipment and instruments.

BENEFITS OF PERFORMING THE TESTS

The benefit for you will be to know the level of your complete functional and anthropometric status as required by the latest recommendations of the scientific community.

By participating in this study you will be able to take part in a physical exercise program, with different types of exercises, in a systematic, programmed and supervised way by qualified physiotherapists. The

sessions will be directed and carried out by physiotherapists specifically trained in therapeutic exercise for the elderly. The realization in small groups will allow us to recommend guidelines, habits and behaviors to follow in order to optimize the way of performing the exercises obtaining the best results and the transfer of the benefits to your daily life.

The information obtained from this research project may contribute to scientific progress and could help other patients in the future. You will not receive any financial benefit for your participation and the release of the data provided, nor will you have any rights to any potential commercial benefits from the discoveries that may be made as a result of the research conducted.

This research study has been approved by the Medicine Ethics Committee of the Salamanca Health Area. The Project is funded under the Art. 83 Project/Contract between the Salamanca City Council (Concejalía de Mayores) and the University of Salamanca.

HANDLING OF YOUR PERSONAL DATA

All information about this study will be stored in coded form, and will be used exclusively for the purposes specified herein. In the event that your data are transferred to other research groups, this will always be done in accordance with current legislation, keeping your data coded, in order to carry out studies related to the objectives of this work, and with the prior authorization of the Ethics Committee of the Salamanca Health Area. In case the objectives of the research work proposed by other research groups are different from those of the present project, a new consent will be requested.

PARTICIPATION AND VOLUNTARY WITHDRAWAL

You are free to decide whether or not to take part in this study, participation is completely voluntary. If you decide to participate, you still have the possibility to withdraw at any time without having to give explanations, and without any penalty or negative consequences for you. If you change your mind about your samples or your data, you have the right to request their destruction or anonymization, through your investigator. However, the data obtained in the analyses carried out up to that point may be used for the purposes requested and may be retained in compliance with the corresponding legal obligations.

DATA PROTECTION AND CONFIDENTIALITY

All information about your results will be treated in the strictest confidence. Your data will be identified by a code, so that it does not include information that can identify you, and only the research team will be able to relate such data to you. The research team assumes responsibility for the protection of personal data. Both your samples and your data will be kept under appropriate security conditions and it is guaranteed that the subjects cannot be identified through means considered reasonable by persons other than those authorized. If the results of the study are likely to be published in scientific journals, no personal data of the participants in this research will be provided at any time. Your personal data will be protected in accordance with the provisions of Organic Law 3/2018, of December 5, 2018, on the Protection of Personal Data and guarantee of digital rights and Regulation (EU) 2016/679 of the European Parliament and of the Council of 27 April 2016 on the Protection of Personal Data and the guarantee of digital rights and Regulation (EU) 2016/679 of the European Parliament and of the Council of 27 April 2016 on the Protection of Personal Data.

Council of 27 April 2016 on Data Protection (GDPR, having the right to access, rectify or cancel your data, and you can limit the processing of data that is incorrect, request a copy or that the data you have provided for the study be transferred to a third party. To exercise your rights, please contact the principal investigator of the study whose details are specified at the end of this document or the Data Protection Officer of our center (dpd.husa@saludcastillayleon.es). You also have the right to contact the Data Protection Agency if you are not satisfied.

Contact details of the research:

Name: Roberto Méndez Sánchez. Email: ro_mendez@usal.es

Phone: 923294590 ext 3201

INFORMED CONSENT

D/D[a] .... with D.N.I. .... in full use of my faculties, freely and voluntarily DECLARE:

That I have requested to participate voluntarily in the GRP organized and financed by the City Council of Salamanca, whose Principal Investigator and responsible is Dr. D. Roberto Méndez Sánchez, physiotherapist and tenured university professor of the University of Salamanca.

That I have read the information on the objectives, methodology, benefits, discomforts and risks derived from my participation in the aforementioned GRP, and that the questions I have been asked personally by Dr. Méndez or his collaborators have been answered in a way that is understandable to me and to my complete satisfaction.

That I have been informed that in order to be able to participate actively in the program, and to achieve the objectives proposed therein, it is necessary for me to undergo a series of preliminary examinations to assess the evolution of the variables to be evaluated.

I declare that I have faithfully and truthfully provided the information about my physical and health condition that could affect my participation in the GRP.

That I am aware that I can refuse to take any or all of the tests and assessments indicated in the information sheet, whenever I wish, and without having to give explanations, and I also understand that the performance of some tests is essential and essential to be included in some activities of the Program.

That I have been assured that the data and information derived from the tests and functional assessments that will be performed during my participation in the GRP will be treated with the maximum confidentiality required by law, that they will not be transferred or given to any person or entity, under any circumstances, nor will they be used publicly for any purpose whatsoever.

Therefore, I hereby give my consent and give my informed consent and authorization to Dr. Roberto Méndez Sánchez and the members of his team that he deems appropriate, to carry out the tests and assessments detailed in this consent.

In Salamanca, at .... from .... from ....

Participant's signature   Name and signature of researcher

Date   Date
