# Supplementary material for: Effect of power training on function and body composition in older women with probable sarcopenia. A study protocol for a randomized controlled trial
Source: PLoS One. 2025 Jan 6;20(1):e0313072. doi: 10.1371/journal.pone.0313072 (PMC11703083; doi:10.1371/journal.pone.0313072)
Supplement: S2 Table — (DOCX) [file pone.0313072.s003.docx]

| **Supplementary file 1.** Standardized measurement of the result variables. | | | |
| --- | --- | --- | --- |
| **5STS** | Performed using an armless folding chair with a seat height of 17 inches (43.2 cm). The time starts when the patient initiates the first squat from the seated position and ends when the patient returns to a seated position after performing all 5 squads (26). | | |
| **HG** | To perform the measurement in a standardised manner, all participants were placed in a standing position, elbow flexed at 90º and neutral pronosupination, and were told: "Squeeze with all your strength for 3 seconds! The test was performed alternately with the left and right hand and the mean was obtained as the valid value (44). | | |
| **SPPB:** | This is a rapid, objective physical function test with three tests. It is scored from 0 to 12 points, each section can be scored up to 4 points according to the marks obtained (31). | Balance | Held for 10s with feet together. Subsequently in a *semitandem* position. The last test had to be held for more than 10 seconds in tandem position. |
|  |  | Gait Speed | The patient stands a few metres before the line where the time starts and is instructed to walk at his or her usual speed until three metres after the line where the time stops (45). The distance over which it was calculated was 4 metres. It was performed three times and the best value was chosen. |
|  |  | 5STS | Same. |
| **TUG** | Consists of measuring the time it takes an individual to get up from a chair, walk a distance of 3 metres, turn around and return to a sitting position, all at normal speed and without assistance. The test was carried out twice and the best mark was chosen, taking care not to use the hands to get up (32). | | |
| **TME2'** | To develop the TME2', the assessed patient will be instructed to walk in the same place where he/she is located, as fast as possible for 2 minutes while raising his/her knees to an intermediate height between the knees and the iliac crests. The test result is defined as the number of steps counted on the right side at the set height in 2 minutes (46). | | |
| **Body weight, ASM, SMI, BMI, %MT, %BF, visceral fat)** | All participants had their weight reduced by 1 kg for clothing. All subjects were properly hydrated before the test. The surface of the BIA was wetted to improve handling and all participants were measured completely barefoot. | | |
| **WC** | Measured with a tape measure at the level of the umbilicus, after a deep breath in and out, without pressure (47). | | |
